# Associations of ADL Disability With Trunk Muscle Mass and Muscle Quality Indicators Measured by Opportunistic Chest Computed Tomography Imaging Among Older Inpatients

**DOI:** 10.3389/fmed.2021.743698

**Published:** 2021-10-28

**Authors:** Xiaofan Jing, Lingling Tan, Hongbo Fu, Ling Yang, Ming Yang

**Affiliations:** ^1^Department of Clinical Nutrition, West China Hospital, Sichuan University, Chengdu, China; ^2^Center of Gerontology and Geriatrics, West China Hospital, Sichuan University, Chengdu, China; ^3^Outpatient Department, West China Hospital, Sichuan University, Chengdu, China; ^4^National Clinical Research Center for Geriatrics, West China Hospital, Sichuan University, Chengdu, China

**Keywords:** muscle depletion, muscle quality, muscle wasting, fat infiltration in muscle, myosteatosis

## Abstract

**Objectives:** Sarcopenia is an important predictor of dependence in activities of daily living (ADL disability); however, the association between muscle quality and ADL disability has not been established. We aimed (1) to assess the feasibility of measuring trunk muscle mass and muscle quality by chest CT images; and (2) to explore the possible associations of ADL disability with these muscle mass and muscle quality indicators among older inpatients.

**Methods:** We included older patients in an acute care ward. ADL disability was defined as the Barthel Index (BI) score ≤ 60 points. Unenhanced chest CT images at the 12^th^ thorax (T12) vertebral level were used to segment skeletal muscle area (SMA) and intermuscular adipose tissue (IMAT) and to measure the mean skeletal muscle radiodensity (SMD). Skeletal muscle index (SMI), the muscle mass indicator, was calculated by SMA (cm^2^)/body height squared (m^2^). The percentage of IMAT (IMAT%) was calculated using the equation: IMAT% = IMAT/(SMA+ IMAT) ×100%. Skeletal muscle radiodensity, IMAT, and IMAT% were the muscle quality indicators. Kendall's tau rank correlation coefficients (τ) were calculated to explore the correlations. Univariate and multivariate logistic regression models were performed to calculate odds ratios (OR) and 95% confidence interval (CI).

**Results:** We included 212 participants. Skeletal muscle index and SMD were positively and significantly associated with the BI score (τ = 0.14 and 0.31, respectively, both *P* < 0.001); whereas IMAT and IMAT% were negatively and significantly associated with the BI score (τ = −0.21, *P* < 0.001; τ = −0.21, *P* < 0.012). After adjusting for confounders, SMI (adjusted OR 1.03, 95% CI 0.97–1.09) was not independently associated with ADL disability; however, SMD (adjusted OR 0.94, 95% CI 0.88–0.99), IMAT (adjusted OR 1.11, 95% CI 1.03–1.20), and IMAT% (adjusted OR 1.09, 95% CI 1.02–1.16) were independently associated with ADL disability. Subgroup analysis found similar results in men; however, none of these indicators were independently associated with ADL disability in women.

**Conclusion:** Trunk muscle quality indicators (SMD, IMAT, and IMAT%) measured by chest CT images, but not SMI, are independently associated with ADL disability in a single-center study population of older inpatients, especially in men. Further research is necessary to validate our findings.

## Introduction

Activities of daily livings (ADLs) are the basic tasks a person must perform to maintain their independence at home. Having difficulty performing ADLs, which is called ADL disability, is associated with an increased risk of morbidity and mortality (1). The prevalence of ADL disability increases with aging and has been reported highly prevalent in older adults, especially in older inpatients (2–4). Activities of daily living disability has become a public health issue in the aging world (5).

Skeletal muscle mass is essential for maintaining physical function and performing ADL (6). Sarcopenia, the loss of muscle mass and muscle function, has been associated with ADL disability (2). Recently, an updated version of the European Working Group on Sarcopenia in Older People (EWGSOP2) (7), the most widely used sarcopenia guideline (8), highlighted that muscle quality was as critically important as muscle mass. Muscle quality refers to “micro- and macroscopic changes in muscle architecture and composition” (9). The EWGSOP2 recommended highly-sensitive imaging tools, such as computed tomography (CT), to assess muscle quality, by measuring intermuscular adipose tissue (IMAT) and skeletal muscle radiodensity (SMD), indicating intramuscular adipose tissue (IntraMAT) (8).

Two recent studies reported that IntraMAT in quadriceps was strongly associated with ADL disability and the incidence of ADL disability in older inpatients (10, 11). However, both studies measured IntraMAT by ultrasound, which is not the “gold” method for measuring muscle quality. In contrast, IMAT measured by CT was not significantly associated with mobility disability and the risk of decline in gait speed in community-dwelling older adults (12). Moreover, most previous studies used abdominal or thigh CT images to assess muscle mass and muscle quality (13). In clinical practice, chest CT scans are far more common than abdominal imaging or thigh imaging, especially among hospitalized patients. Moreover, ADL disability is prevalent among older inpatients and correlates with poor health and financial outcomes among this population (14). Therefore, we aimed (1) to assess the feasibility of measuring trunk muscle mass and muscle quality by chest CT images; and (2) to explore the possible associations of ADL disability with these muscle mass and muscle quality indicators among older inpatients.

## Methods

### Study Design and Participants

We conducted a cross-sectional study from January 2018 to December 2020. We consecutively invited older patients (aged 65 years and over) who were referred to the Center of Gerontology and Geriatrics, West China Hospital, to participate in this study. We excluded patients with any of the following conditions: (1) acute stroke; (2) New York Heart Association Classification Class IV heart failure; (3) severe respiratory failure (hypoxia with a PaO_2_/FiO_2_ ratio less than 300 mmHg) (15); (4) medical history of dementia, delirium, depression, anxiety, or hemodialysis; (5) trauma, surgery, or bone fracture within 3 months prior to enrollment; (6) refusal to sign the consent; (7) terminal diseases; (8) without chest CT images within 48 h after admission; (9) low-quality CT images or any anatomical distortion (e.g., chest wall edema or pleural effusion) or loss of any muscle mass area on CT images. All participants signed the informed consent form prior to participation. The study protocol was approved by the Biomedical Ethics Committee of West China Hospital, Sichuan University.

### ADL Measurement

We applied the Barthel Index (BI) (16), a classic self-reported questionnaire, to assess ADL for each participant. The BI includes 10 items: bathing, toilet action, bowel continence, bladder continence, dressing, feeding, grooming, walking on a surface, going up and downstairs, and moving from a chair to a bed and from a bed to a chair. The BI score ranges from 0 points (total dependence) to 100 points (complete independence). The lower the BI score, the worse the ability to perform ADL tasks (16). As previously reported (16, 17), we defined ADL disability as the BI score ≤ 60 points.

### Measurements of Muscle Mass and Muscle Quality Indicators

Each participant received chest CT scans within 48 h after admission for acute respiratory infection, chest pain, or other reasons. The CT scans were performed using a 16-slice spiral CT scanner (Brilliance; Philips Healthcare, Ohio, USA) with a 5-mm slice thickness. In this study, we applied a dedicated segmentation software (Mimics version 21.0; Materialise, Leuven, Belgium) to analyze unenhanced cross-sectional CT images at the 12^th^ thorax (T12) vertebral level.

Using a single CT image, skeletal muscle area (SMA) was segmented according to muscle tissue thresholds [−29 to 150 Hounsfield unit (HU)] (18). The HU scale reflects the density of tissue on a CT scan (19). In this study, SMA includes all skeletal muscle visible at the T12 vertebral level (i.e., *erector spinae, latissimus dorsi, rectus abdominis, obliquus externus, internus abdominis*, and *internal and external intercostal muscles*). According to previous studies (20, 21), we calculated skeletal muscle index (SMI) using the following equation: SMI = SMA (cm^2^)/body height squared (m^2^) to adjust for the impact of body size. A higher SMI indicates more skeletal muscle.

For muscle quality evaluation, we applied Mimics software to calculate the mean SMD of all skeletal muscles at the T12 vertebral level. In cases of lower SMD, there will be a larger percentage of IntraMAT (22), indicating the lower quality of skeletal muscle (23). Moreover, IMAT was assessed by identifying visible adipose tissue within muscle fascia at the T12 vertebral level according to fat tissue thresholds (−30 to−190 HU) (24, 25). Higher IMAT indicates poorer muscle quality. The percentage of IMAT (IMAT%) was calculated using the equation: IMAT% = IMAT (cm^2^)/(SMA (cm^2^) + IMAT (cm^2^)) ×100% (26).

### Measurements of Covariates

Trained staff assessed the nutrition status of the participants within 48 h after admission using the mini nutritional assessment short form (MNA-SF), a validated screening tool for identifying malnutrition in older adults (27). The score of MNA-SF ranges from 0 (best) to 14 points (worst). Moreover, the following information was obtained from the Hospital Information System: age, sex, body weight, height, body mass index, comorbidities (hypertension, cardiovascular disease, any type of cancer, diabetes, chronic obstructive pulmonary disease, chronic kidney disease, acute infection), albumin, and hemoglobin.

### Statistical Analysis

The statistical analyses were performed in R version 3.5.1 (R Foundation for Statistical Computing, Vienna, Austria) and SPSS software 26.0 (IBM SPSS Inc., New York, US). A *p*-value <0.05 indicates statistical significance. The distribution of continuous data was evaluated using the Shapiro-Wilk test. Continuous data are presented as mean and standard deviation or median and interquartile range where appropriate. Categorical data are presented as numbers and percentages. Group differences were compared using independent samples *t*-test, Mann-Whitney U-test, or Chi-squared test. Kendall's tau rank correlation coefficients (τ) were calculated to explore the correlations of age, BMI, MNA-SF score, BI score, albumin, and hemoglobin with SMI, SMD, IMAT, and IMAT%. The correlation coefficients are considered as high, moderate, or low when τ is >0.5, 0.3–0.5, or <0.3, respectively (22). Univariate logistic regression was performed to explore the association of different variables with ADL disability. Multivariate logistic regression analysis was performed to assess the association of SMI, SMD, IMAT, and IMAT% with ADL disability with adjustment for age, sex, cardiovascular disease, diabetes, MNA-SF score, and albumin. The results of the logistic regression models are presented as odds ratios (ORs) with 95% confidence intervals (CIs). Based on the significant differences regarding SMI, SMD, IMAT, and IMAT% between men and women found in data exploration using independent samples *t*-tests and Mann-Whitney U-tests, the main results were further stratified by sex.

## Results

### Patient Characteristics

Initially, 221 subjects were enrolled, but nine patients were excluded because we were unable to segment their CT images due to chest wall edema (*n* = 2), pleural effusion (*n* = 6), or loss of muscle mass on the CT image (*n* = 1). Thus, we successfully measured muscle mass and muscle quality indicators in 96% (212/221) participants.

We finally included 212 older inpatients (162 men and 50 women) in the analysis. The characteristics of the participants are presented in Table 1. Men were significantly older than women (median: 85.0 and 81.0 years of age, respectively, *P* = 0.001). Not surprisingly, men had more skeletal muscle than women (mean SMI: 27.9 and 23.5 cm^2^/m^2^, respectively, *P* < 0.001). However, there was no significant difference between men and women regarding skeletal muscle quality indicators (i.e., SMD, IMAT, and IMAT%). Supplementary Table 1 shows the characteristics of the participants according to ADL disability. Patients with ADL disability were significantly older than those without (median: 87.0 and 82.0 years of age, respectively, *p* < 0.001). Moreover, patients with ADL disability were at higher risk of malnutrition than those without (median MNA-SF score: 7.0 vs. 10.0 points, *P* < 0.001; mean serum albumin: 34.9 vs. 38.7 g/L; *P* < 0.001).

**Table 1:** Characteristics of the study population (*n* = 212).

	**Men (*n* = 162)**	**Women (*n* = 50)**	***P*-value**
Age, years^*^	85.0 (11.3)	81.0 (10.8)	0.001
Weight, kg	60.5 (10.1)	55.0 (12.1)	0.001
Height, cm^*^	167.0 (9.0)	155.0 (9.0)	<0.001
Body mass index, kg/m^2^	23.8 (3.9)	22.2 (7.6)	0.187
Comorbidities (%)			
Hypertension	77 (47.5)	24 (48.0)	0.954
Cardiovascular disease	53 (32.7)	15 (30.0)	0.719
Any type of cancer	53 (32.7)	11 (22.0)	0.149
Diabetes	95 (58.6)	36 (72.0)	0.089
Chronic obstructive pulmonary disease	16 (9.9)	8 (16.0)	0.232
Chronic kidney disease	23 (14.2)	11 (22.0)	0.189
Acute infection	68 (42.0)	13 (26.0)	0.042
MNA-SF score^*^	9.0 (5.0)	9.0 (6.0)	0.971
Barthel Index score^*^	85.0 (50.0)	80.0 (41.3)	0.698
Hemoglobin, g/L^*^	119.5 (31.0)	117.0 (32.0)	0.219
Albumin, g/L	36.3 (5.3)	39.3 (5.1)	<0.001
Body composition based on CT image			
SMI, cm^2^/m^2^	27.9 (7.0)	23.5 (6.3)	<0.001
SMD, HU^*^	33.4 (9.9)	31.6 (10.5)	0.244
IMAT, cm^2^*^^	6.2 (6.3)	5.0 (6.0)	0.648
IMAT%, %^*^	8.1 (7.6)	9.4 (10.3)	0.050

*Data presented as n (percentage) or mean (standard deviation) if not specified. IMAT, intermuscular adipose tissue; IMAT%, percentage of IMAT; MNA-SF: mini nutritional assessment short form; SMA, skeletal muscle area; SMD, skeletal muscle radiodensity, SMI, skeletal muscle index*.

* *Data presented as median and interquartile range*.

### Associations of Muscle Mass and Muscle Quality Indicates With Age and BI Score

**Figure 1:**
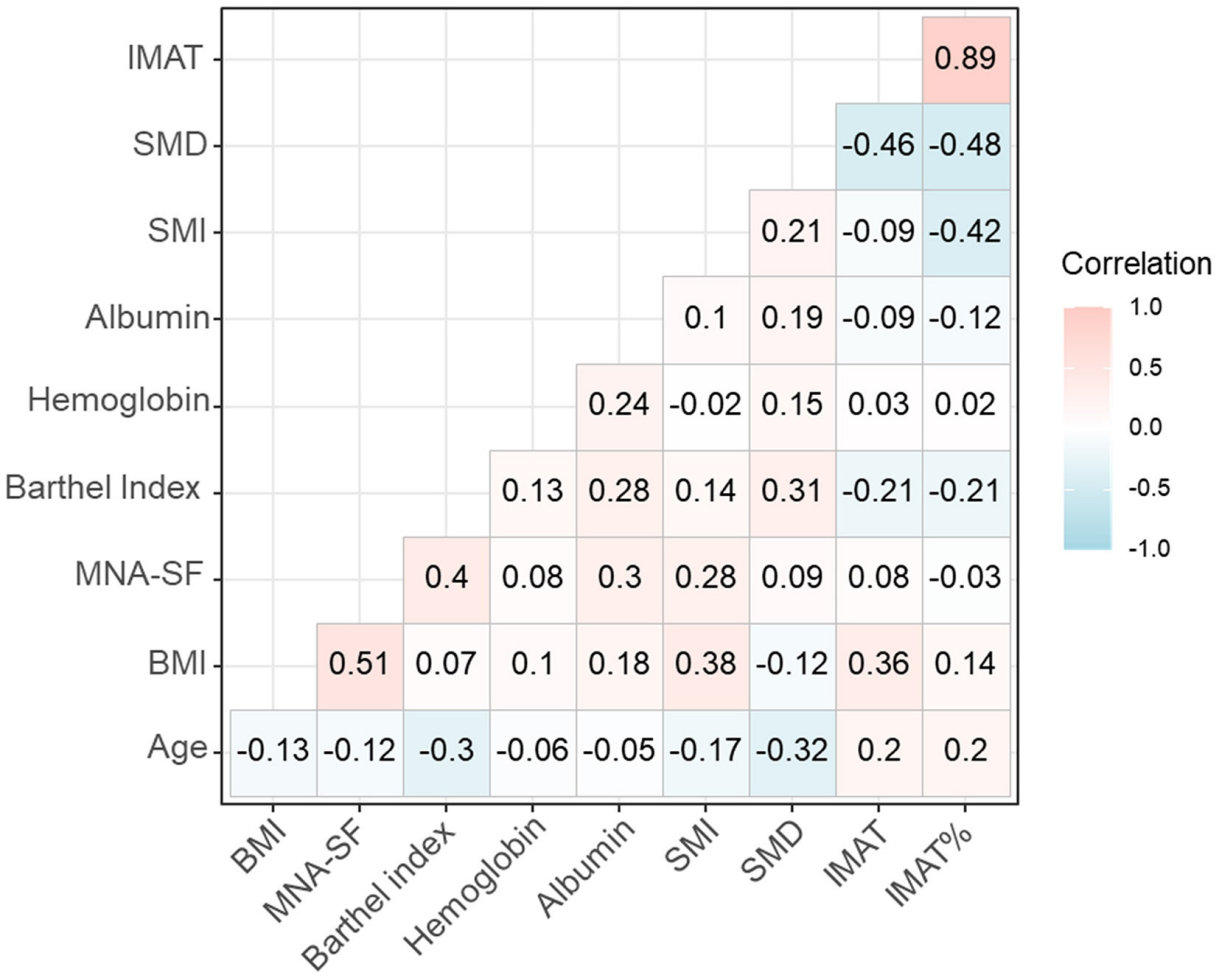
The correlation coefficients of SMI, SMD, IMAT, and IMAT% with other variables. BMI, body mass index; IMAT, intermuscular adipose tissue; IMAT%, percentage of intermuscular adipose tissue; MNA-SF, mini nutritional assessment short form; SMD, skeletal muscle radiodensity; SMI, skeletal muscle index.

Figure 1 shows the correlation coefficients of SMI, SMD, IMAT, and IMAT% with other variables. SMI was positively and significantly associated with the BI score (τ = 0.14, *P* < 0.001), but negatively and significantly associated with age (τ = −0.17, *P* < 0.001). Similarly, SMD was positively and significantly associated with the BI score (τ = 0.31, *P* < 0.001), but negatively and significantly associated with age (τ = −0.17, *P* < 0.001). In contrast, IMAT was negatively and significantly associated with the BI score (τ = −0.21, *P* < 0.001), but positively and significantly associated with age (τ = 0.20, *P* = 0.001). IMAT% was also negatively and significantly associated with the BI score (τ = −0.21, *P* = 0.012), but positively and significantly associated with age (τ = 0.20, *P* = 0.011).

### Associations of Muscle Mass and Muscle Quality Indicates With ADL Disability

**Figure 2:**
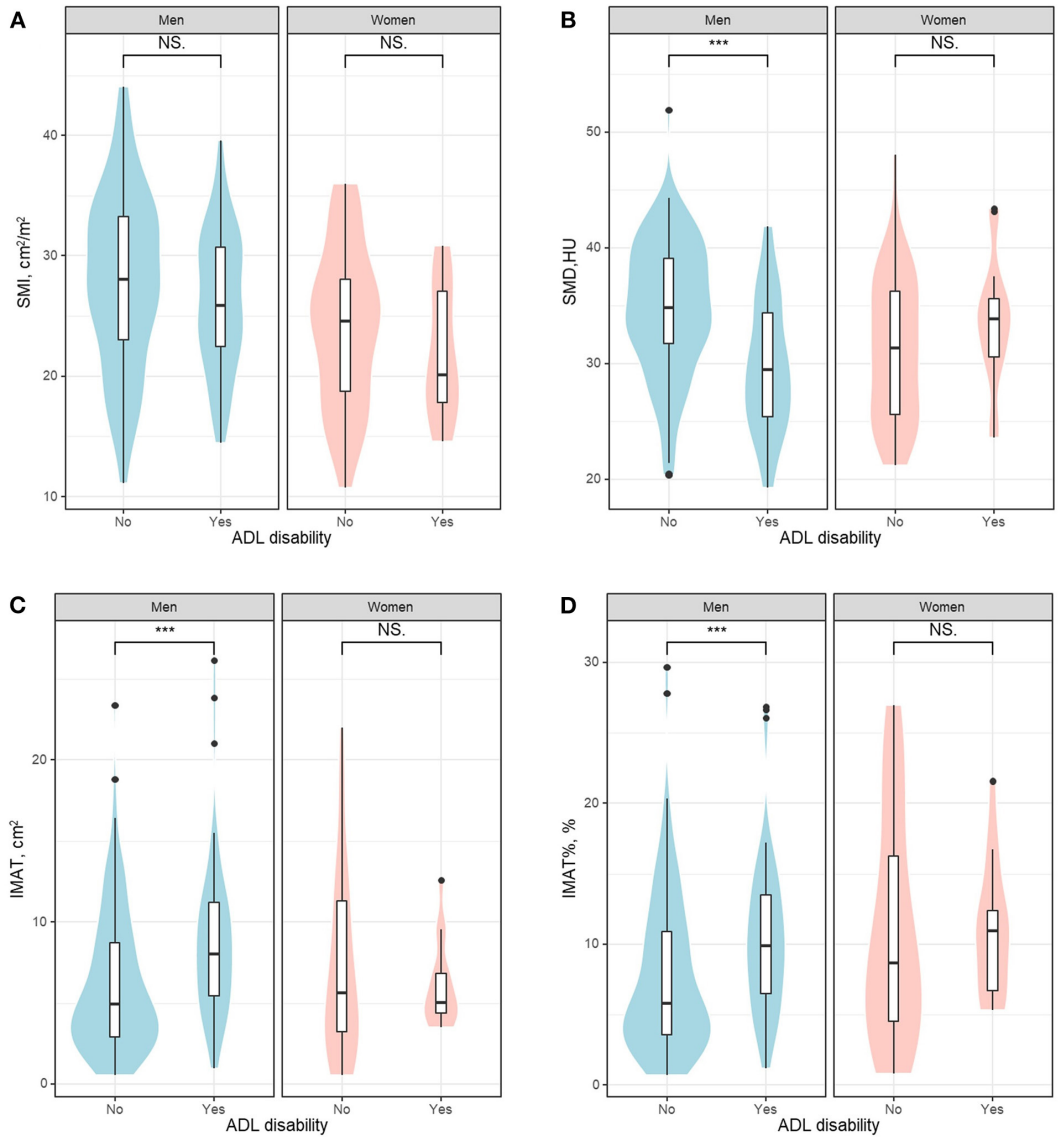
The violin plot and boxplot of SMI **(A)**, SMD **(B)**, IMAT **(C)**, and IMAT% **(D)** between patients with or without ADL disability. ADL, activities of daily living; HU, Hounsfield unit; IMAT, intermuscular adipose tissue; IMAT%, percentage of intermuscular adipose tissue; NS, not significant; SMD, skeletal muscle radiodensity; SMI, skeletal muscle index. ***indicates *P* < 0.001.

Figure 2 shows the violin plot and boxplot of SMI, SMD, IMAT, and IMAT% between patients with or without ADL disability. In both men and women, patients with ADL disability appeared to have higher SMI (indicating more muscle mass) than those without ADL disability but the group differences were not statistically significant (Figure 2A). Compared with their counterparts, men with ADL disability had significantly lower SMD but significantly higher IMAT and IMAT% (Figures 2B–D, all *p* < 0.001). Among women, there was no significant difference between groups regarding SMD, IMAT, and IMAT% (Figures 2B–D). Typical CT images of patients with or without ADL disability are shown in Supplementary Figure 1.

Table 2 shows the univariate logistic analyses to explore the association between ADL disability and different variables. As shown in Table 2, SMI was not associated with ADL disability (OR 0.97, 95% CI 0.93–1.01); however, muscle quality indicators were significantly associated with ADL disability (SMD: OR 0.91, 95% CI 0.87–0.96; IMAT: OR 1.08, 95% CI 1.02–1.15; and IMAT%: OR 1.05, 95% CI 1.01–1.10). Subgroup analyses indicated that SMI was not associated with ADL disability in both men and women (Table 2). Muscle quality indicators (SMD, IMAT, and IMAT%) were significantly associated with ADL disability in men but not in women (Table 2).

**Table 2:** Univariate logistic analyses to explore the association between ADL disability and different variables.

	**Total (*****n*** **=** **212)**		**Men (*****n*** **=** **162)**		**Women (*****n*** **=** **50)**	
	**OR (95% CI)**	***P*-value**	**OR (95% CI)**	***P*-value**	**OR (95% CI)**	***P*-value**
Age, years	1.09 (1.05–1.14)	<0.001	1.10 (1.05–1.16)	<0.001	1.07 (0.97–1.18)	0.158
Women	0.74 (0.37–1.48)	0.389	–	–	–	–
Body mass index, kg/m^2^	0.95 (0.88–1.03)	0.187	1.00 (0.91–1.10)	0.945	0.86 (0.74–1.00)	0.051
Comorbidities						
Hypertension	1.49 (0.84–2.65)	0.175	1.45 (0.76–2.78)	0.264	1.67 (0.48–5.79)	0.422
Cardiovascular disease	1.70 (0.93–3.10)	0.084	1.77 (0.90–3.48)	0.101	1.44 (0.39–5.38)	0.583
Any type of cancer	1.21 (0.65–2.23)	0.553	1.23 (0.62–2.44)	0.555	0.96 (0.21–4.27)	0.951
Diabetes	1.71 (0.93–3.14)	0.086	2.05 (1.03–4.07)	0.040	0.96 (0.24–3.78)	0.955
Chronic obstructive pulmonary disease	1.52 (0.64–3.63)	0.341	1.54 (0.54–4.38)	0.419	1.69 (0.35–8.28)	0.517
Chronic kidney disease	1.31 (0.62–2.81)	0.481	1.26 (0.51–3.12)	0.620	1.66 (0.40–6.88)	0.487
Acute infection	1.46 (0.82–2.62)	0.202	1.48 (0.77–2.84)	0.243	1.20 (0.30–4.79)	0.796
MNA-SF score	0.72 (0.65–0.81)	<0.001	0.74 (0.65–0.84)	<0.001	0.66 (0.50–0.86)	0.003
Hemoglobin, g/L	0.99 (0.98–1.00)	0.202	0.99 (0.98–1.01)	0.346	0.98 (0.95–1.01)	0.980
Albumin, g/L	0.89 (0.84–0.94)	<0.001	0.90 (0.84–0.96)	0.002	0.82 (0.71–0.96)	0.013
Body composition based on CT image						
SMI, cm^2^/m^2^	0.97 (0.93–1.01)	0.121	0.97 (0.92–1.01)	0.140	0.94 (0.85–1.05)	0.269
SMD, HU	0.91 (0.87–0.96)	<0.001	0.87 (0.81–0.92)	<0.001	1.06 (0.96–1.18)	0.260
IMAT, cm^2^	1.08 (1.02–1.15)	0.009	1.13 (1.05–1.22)	0.001	0.95 (0.84–1.09)	0.488
IMAT%, %	1.05 (1.01–1.10)	0.026	1.09 (1.03–1.16)	0.005	1.00 (0.92–1.09)	0.990

*HU, Hounsfield unit; IMAT, intermuscular adipose tissue; IMAT%, percentage of IMAT; MNA-SF, mini nutritional assessment short form; SMA, skeletal muscle area; SMD, skeletal muscle radiodensity, SMI, skeletal muscle index*.

Table 3 shows the multivariate logistic analyses to explore the association of ADL disability with skeletal muscle mass and muscle quality indicators. After adjustment for potential confounders, SMI (adjusted OR 1.03, 95% CI 0.97–1.09) was not independently associated with ADL disability; however, SMD (adjusted OR 0.94, 95% CI 0.88–0.99), IMAT (adjusted OR 1.11, 95% CI 1.03–1.20), and IMAT% (adjusted OR 1.09, 95% CI 1.02–1.16) were independently associated with ADL disability. Subgroup analysis found similar results in men; however, none of these indicators were independently associated with ADL disability in women (Table 3).

**Table 3:** Multivariate logistic analyses to explore the association of ADL disability with skeletal muscle mass and muscle quality indicators measured by chest CT image^*^.

	**Total (*****n*** **=** **212)**		**Men (*****n*** **=** **162)**		**Women (*****n*** **=** **50)**	
	**OR (95% CI)**	***P*-value**	**OR (95% CI)**	***P*-value**	**OR (95% CI)**	***P*-value**
SMI, cm^2^/m^2^	1.03 (0.97–1.09)	0.320	1.03 (0.97–1.10)	0.312	0.98 (0.83–1.15)	0.976
SMD, HU	0.94 (0.88–0.99)	0.035	0.89 (0.83–0.96)	0.002	1.01 (0.87–1.18)	0.916
IMAT, cm^2^	1.11 (1.03–1.20)	0.007	1.15 (1.05–1.26)	0.003	1.14 (0.85–1.53)	0.387
IMAT%, %	1.09 (1.02–1.16)	0.005	1.10 (1.05–1.17)	0.001	1.07 (0.91–1.26)	0.412

*CI, confidence interval; HU, Hounsfield unit; IMAT, intermuscular adipose tissue; IMAT%, percentage of IMAT; MNA-SF, mini nutritional assessment short form; OR, odds ratio; SMA, skeletal muscle area; SMD, skeletal muscle radiodensity; SMI, skeletal muscle index*.

* *These indicators were included in the multivariate logistic models independently, which adjusted for age, cardiovascular disease, diabetes, MNA-SF score, and albumin. In the whole sample, these multivariate logistic models further adjusted for sex*.

## Discussion

Our study was the first to explore the associations of ADL disability with trunk muscle mass indicator (SMI) and muscle quality indicators (SMD, IMAT, and IMAT%) that were measured by opportunistic chest CT images among older patients in an acute care ward. We found that increased fat infiltration in trunk muscles at the T12 vertebral level was independently associated with ADL disability, especially in men. However, trunk muscle mass at the T12 vertebral level was not associated with ADL disability in either men or women.

Our study indicates that it is feasible to segment chest CT images to assess muscle mass and quality. We successfully extracted the relevant data from 96% CT images. Computed Tomography image analysis requires specialized software and trained personnel. In this study, we applied Mimics software, which has been successfully applied in other populations (28). In a previous study, segmentation software programs (FatSeg, OsiriX, ImageJ, and sliceOmatic) showed excellent agreement in measuring muscle mass indicators (29). However, the comparison of Mimics with other software programs that measure muscle mass and quality indicators needs to be further explored.

Only two previous studies addressed the associations of muscle mass and muscle quality with ADL in older inpatients (10, 11). Based on a cross-sectional study with 371 older inpatients, Akazawa et al. (11) reported that increased IntraMAT of the quadriceps was more strongly associated with declines in ADL than the loss of muscle mass. Most recently, the same team conducted a prospective cohort study with 404 older inpatients, and they found that increased IntraMAT of the quadriceps at admission was more strongly associated with worse recovery of ADL than the loss of muscle mass (10). Notably, both studies used ultrasound to measure muscle thickness and measured echo intensity as the surrogates of muscle mass and IntraMAT, respectively. Our results were in line with their findings, but we used chest CT images to assess muscle mass and muscle quality. Computed Tomography is one of the “gold” methods to measure muscle mass and fat infiltration in muscle, and it is supposed to be better than ultrasound in this field.

Unlike the two previous studies (10, 11), we measured trunk muscles at the T12 vertebral level instead of thigh muscles. Research on the relationship of muscle composition with physical function has focused primarily on the thigh muscle area (30). However, there was evidence that attenuation of trunk muscles (i.e., decreased SMD) explains a greater proportion of variance in lower extremity physical function than an attenuation of thigh muscles, highlighting the importance of trunk muscles for physical performance (31). These findings emphasized the importance of trunk muscle quality in physical function and ADL in older adults, implying that improving trunk muscle quality might improve functional status in older inpatients.

Sarcopenia has been proven as an important predictor of ADL among different study populations (2, 32–34). In these studies, sarcopenia was defined by the EWGOSP criteria, including both low muscle mass and decreased muscle function. However, there is controversy as to whether low muscle mass alone is associated with ADL impairments or not. For example, a recent meta-analysis included nine relevant studies and summarized that low muscle mass was positively associated with ADL disability in five of the nine studies. Our study indicated that although SMI was positively and significantly associated with the BI score, it was no longer independently associated with ADL disability after adjustment for confounders. Similarly, in a recent cross-sectional study conducted in 316 community-dwelling volunteers, Wang et al. (35) reported that SMD, but not low muscle mass, correlated well with handgrip strength and physical performance. Moreover, Perez-Sousa et al. (36) reported that gait speed, a surrogate of physical performance, mediated the association between low muscle mass and ADL disability in 19,705 community-dwelling older adults. These findings highlighted the crucial role of muscle function in the definition of sarcopenia when exploring sarcopenia's relationship to ADL.

Myosteatosis (i.e., excessive fat infiltration in muscle) is now considered a different disease from sarcopenia. Both low SMD and high IMAT indicate myosteatosis. However, in this study, we did not report the association between myosteatosis and ADL disability. This is because there is currently neither a well-established diagnosis modality nor diagnostic cutoffs of SMD or IMAT to define myosteatosis. For example, a recent systematic review found that 32 different cutoffs of SMD were used to define myosteatosis across 73 included studies (37). Additionally, previous studies regarding myosteatosis primarily focus on malignant diseases (55 out of 73 studies), liver diseases (seven studies), and cardiovascular diseases (five studies) (37). The prevalence of myosteatosis (either defined by increased IMAT or decreased SMD) was supposed to rise with advancing age; however, studies examining myosteatosis in older adults are relatively rare (38, 39). The opportunistic utility of chest CT images to diagnose myosteatosis may facilitate relevant studies in older populations.

## Limitations

First, our study was of cross-sectional design. Thus, we could not determine the causal relationship of trunk muscle mass or muscle quality with ADL disability. Second, the sample size of our study was relatively small, especially for women. This might partly explain why the associations of SMI and IMAT with ADL disability were not statistically significant in women. Third, we did not assess some important confounders, such as depression and cognitive function. However, we excluded patients with medical records of dementia and depression. Fourth, this is a cross-sectional study conducted in a single center; therefore, the representation of our sample was limited. Fifth, we dichotomized the BI using the previously reported cutoff to define ADL disability, and statistical information might be lost in the process. Last, we did not test muscle strength and physical performance in this study, which are expected to be associated with both muscle mass and muscle quality.

## Clinical Implications

The opportunistic utility of chest CT images has been increasing to identify other diseases, such as chronic obstructive pulmonary disease and osteoporosis (40, 41). Our study implies the potential value of opportunistic usage of chest CT images to assess loss of muscle mass (a key component of sarcopenia) and myosteatosis among older adults in both clinical practice and research. A recent systematic review demonstrated that most of the previous studies (66 out of 70) used abdominal CT to assess myosteatosis and only one study used chest CT (37). Nevertheless, chest CT is far more frequently used than abdominal CT in clinical practice and has been routinely used in health screening (13). Therefore, there would be a great opportunity to use chest CT images to analyze the quantity and quality of skeletal muscle in sarcopenia and myosteatosis research in the future.

Although muscle quality indicators were associated with ADL disability in our study population, they did not seem to be strong, and further study is necessary. Additionally, further research is needed to determine how these muscle quality indicators (and their combination with muscle function) correlate to diverse clinical outcomes (such as ADL disability and quality of life) among different populations.

## Conclusion

Trunk muscle quality indicators (SMD, IMAT, and IMAT%) measured by chest CT images at the T12 vertebral level were independently associated with ADL disability in a single-center study population of older inpatients, especially in men. Despite the positive and significant association between the SMI and the BI score, the SMI was not independently associated with ADL disability after controlling for confounders. Our study supports the opportunistic utility of chest CT images for assessing muscle mass and muscle quality in older inpatients. More longitudinal studies with large sample sizes in different study populations are required to validate our findings and to determine the diagnostic cutoffs of SMI, SMD, IMAT, and IMAT% for defining low muscle mass and myosteatosis.

## Data Availability Statement

The raw data supporting the conclusions of this article will be made available by the authors, without undue reservation.

## Ethics Statement

The studies involving human participants were reviewed and approved by Biomedical Ethics Committee of West China Hospital, Sichuan University. The patients/participants provided their written informed consent to participate in this study.

## Author Contributions

MY: conception and design of study and statistical analysis. LT: imaging data analysis. XJ and MY: drafting the manuscript. All authors: acquisition, analysis, and interpretation of data and critical revision of the manuscript for important intellectual content.

## Funding

This work was supported by the K&D Program of the Sichuan Science and Technology Department (Grant number: 2020YFS0573). The sponsors played no role in the design, methods, data collection, analysis, or preparation of this work.

## Conflict of Interest

The authors declare that the research was conducted in the absence of any commercial or financial relationships that could be construed as a potential conflict of interest.

## Publisher's Note

All claims expressed in this article are solely those of the authors and do not necessarily represent those of their affiliated organizations, or those of the publisher, the editors and the reviewers. Any product that may be evaluated in this article, or claim that may be made by its manufacturer, is not guaranteed or endorsed by the publisher.
