# Supplementary material for: Associations of ADL Disability With Trunk Muscle Mass and Muscle Quality Indicators Measured by Opportunistic Chest Computed Tomography Imaging Among Older Inpatients
Source: Front Med (Lausanne). 2021 Oct 28;8:743698. doi: 10.3389/fmed.2021.743698 (PMC8581194; doi:10.3389/fmed.2021.743698)
Supplement: Supplementary file 1 [file Table_1.DOCX]

**Supplementary Table 1. Characteristics of the study population according to ADL status (n=212)**

|  | Without ADL disability  (n=142) | With ADL disability  (n=70) | P-value |
| --- | --- | --- | --- |
| Age, years ^*^ | 82.0 (13.0) | 87.0 (9.0) | <0.001 |
| Women | 36 (25.4) | 14 (20.0) | 0.388 |
| Body mass index, kg/m^2^ | 22.1 (5.1) | 21.6 (4.4) | 0.294 |
| Comorbidities (%) |  |  |  |
| Hypertension | 63 (44.4) | 38 (54.3) | 0.174 |
| Cardiovascular disease | 40 (28.2) | 28 (40.0) | 0.083 |
| Any type of cancer | 41 (28.9) | 23 (32.9) | 0.552 |
| Diabetes | 82 (57.7) | 49 (70.0) | 0.084 |
| Chronic obstructive pulmonary disease | 14 (9.9) | 10 (14.3) | 0.339 |
| Chronic kidney disease | 21 (14.8) | 13 (18.6) | 0.480 |
| Acute infection | 50 (35.2) | 31 (44.3) | 0.201 |
| MNA-SF score ^*^ | 10.0 (4.0) | 7.0 (4.0) | <0.001 |
| Hemoglobin, g/L ^*^ | 123.0 (33.0) | 115.0 (25.0) | 0.106 |
| Albumin, g/L | 38.7 (5.7) | 34.9 (5.8) | <0.001 |

Data presented as n (percentage) or mean (standard deviation) if not specified.

Abbreviations: IMAT: intermuscular adipose tissue; IMAT%: percentage of IMAT; MNA-SF: mini nutritional assessment short form; SMA: skeletal muscle area; SMD: skeletal muscle radiodensity, SMI: skeletal muscle index.

^*^ Data presented as median and interquartile range.
