# Supplementary material for: Associations of ADL Disability With Trunk Muscle Mass and Muscle Quality Indicators Measured by Opportunistic Chest Computed Tomography Imaging Among Older Inpatients
Source: Front Med (Lausanne). 2021 Oct 28;8:743698. doi: 10.3389/fmed.2021.743698 (PMC8581194; doi:10.3389/fmed.2021.743698)
Supplement: Supplementary file 2 [file Image_1.pdf]

BI score: 100 points

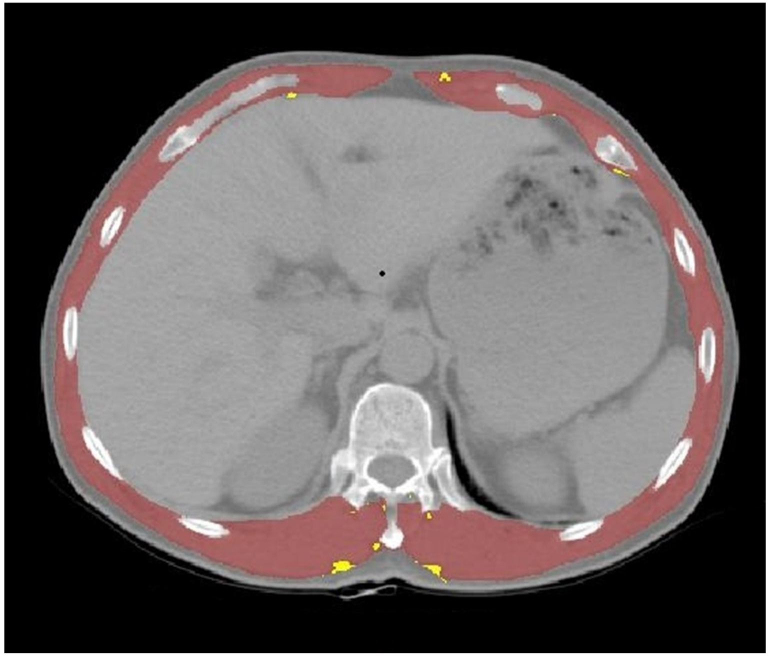

Male, body mass index = 25.8 kg/m^2 , age = 82 years

- SMI = 42.08 cm^2/m^2
- SMD = 42.90 HU
- IMAT = 2.82 cm^2
- IMAT%= 2.55%

BI score: 50 points

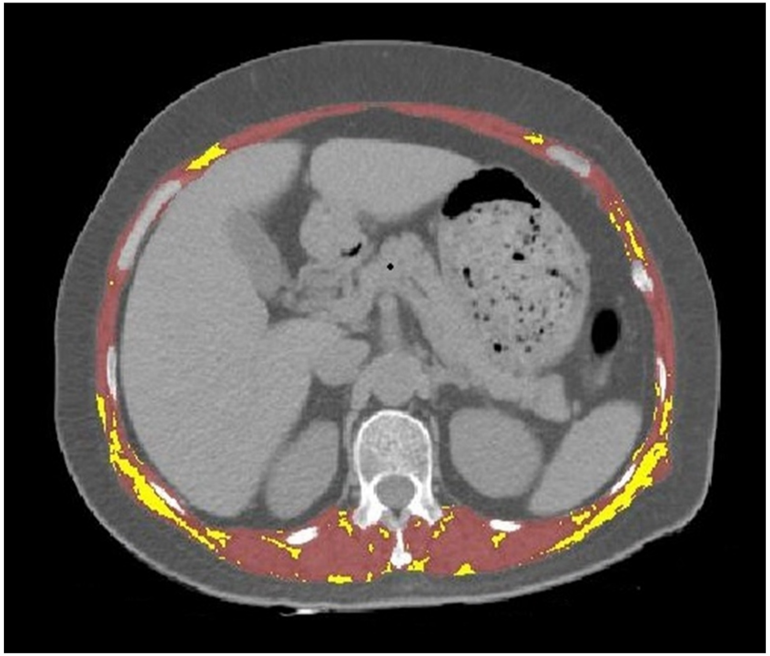

Male, body mass index = 28.6 kg/m^2 , age = 90 years

- SMI = 22.45 cm^2/m^2
- SMD = 19.26 HU
- IMAT = 21.05 cm^2
- IMAT%= 26.09%

Supplementary Figure 1. Typical CT images of patients with or without ADL disability.
BI: Barthel Index; HU: Hounsfield Unit; IMAT: intermuscular adipose tissue; IMAT%: percentage of intermuscular adipose tissue; SMD: skeletal muscle radiodensity; SMI: skeletal muscle index.
